# Concerns about LUNG-SAFE: response to the letter to the Editor of *Critical Care* by Bellani et al.

**DOI:** 10.1186/s13054-016-1477-0

**Published:** 2016-09-17

**Authors:** Jesús Villar, Robert M. Kacmarek

**Affiliations:** 1 CIBER de Enfermedades Respiratorias, Instituto de Salud Carlos III, Madrid, Spain; 2 Multidisciplinary Organ Dysfunction Evaluation Research Network, Research Unit, Hospital Universitario Dr. Negrin, Las Palmas de Gran Canaria, Spain; 3 Keenan Research Center for Biomedical Science at the Li Ka Shing Knowledge Institute, St. Michael’s Hospital, Toronto, Canada; 4 Department of Respiratory Care, Massachusetts General Hospital, Boston, MA USA; 5 Department of Anesthesia, Harvard University, Boston, MA USA

We appreciate the opportunity to respond to the letter by Bellani et al. regarding our commentary on LUNG-SAFE. They expressed concern regarding the four potential biases we raised about their study.

First, we were concerned with the use of the Berlin Criteria to classify patients with ARDS. They indicated that there was no need to validate these criteria. However, these criteria were developed by consensus and have never been validated. All attempts to validate the criteria have failed, indicating that the criteria could not identify patients based on severity with increasing mortality [1, 2].

Second, we indicated that patients with ARDS need to meet the criteria for at least 24 hours. We have demonstrated previously that patients classified as severe ARDS at onset may not even meet the criteria for mild ARDS 24 hours later [3, 4]. There is no evidence which would indicate that the severe, lung inflammatory response that is a hallmark of ARDS on autopsy [5] resolves within a 24-hour period. Without longitudinal data meeting ARDS criteria, it is highly likely that many of these patients simply developed atelectasis.

Third, we agree the authors did not directly state that the incidence of ARDS they identified was over a full 1-year period. However, the implication presented, we believe, would lead the naïve reader to easily interpret these data as implying a global incidence of ARDS.

Finally, the authors state that “nonrecruiting sites” were eliminated. We interpreted this as sites without ARDS patients during the 4-week study period. This is a finding we consider highly plausible but to be adding bias if they were eliminated. We did not even consider that 207 ICUs would be eliminated because there were no mechanically ventilated patients in those ICUs during the 4-week winter study period. A total of 666 ICUs registered to be part of the study but only patients from 459 ICUs were included.

We commend the authors on performing a very difficult and enlightening study but have ongoing concerns with the utility of the Berlin Criteria as the definition for ARDS. The continued failure for validating these criteria may provide the medical community with a highly inflated perception of the incidence of ARDS. We believe the data indicate that a definition with a more precise, standardized ventilator setting requiring criteria to be present over a longitudinal period would provide a much more accurate indication of the incidence of ARDS.

## Abbreviations

ARDS: Acute respiratory distress syndrome
LUNG-SAFE: Large Observational Study to Understand the Global Impact of Severe Acute Respiratory Failure
